# Individualizing Workplace Learning with Digital Technologies: New Learning Cultures in Swiss Apprenticeship Training

**DOI:** 10.1007/978-3-030-55878-9_7

**Published:** 2020-07-15

**Authors:** Antje Barabasch, Anna Keller

**Affiliations:** 1 Learning, Design and Technology / UNESCO Deputy Chair of Data Science in Higher Education Learning and Teaching, University of Mannheim / Curtin University, Mannheim / Perth, Germany; 2 Department of Education and Social Sciences, University of Cologne, Köln, Germany; 3 Learning, Design & Technology, University of Mannheim, Mannheim, Germany; 4 Human Sciences Faculty, University of Cologne, Cologne, Germany; Eidgenössisches Hochschulinstitut Für Berufsbildung (EHB), Zollikofen, Switzerland

**Keywords:** Workplace learning, Vocational education and training, Switzerland, Digital technologies, Case study

## Abstract

Vocational education and training (VET) at Swiss enterprises includes the work with various digital technologies. They ease administration of work hours, work tasks, evaluations or log book remarks; they support communication with peers, co-workers and trainers; and they come in handy for idea development and creative work. Overarching trends in terms of changing learning cultures in apprenticeship training, such as individualization, flexibilization, self-organized learning, project work or coaching, support the introduction of these technologies and also benefit from them. Based on three in-depth case studies, results on the usage and impact of digital technologies will be presented. This chapter addresses the following research questions: Which digital tools are used in workplace training? What are potential benefits and extended justifications for the use of digital tools? How are modern learning cultures impacting the use of digital tools? We will outline how and where digital technologies are used at the workplace in apprenticeship training, why management has introduced them and how apprentices and their trainers benefit from it. Based on our findings, we will draw conclusions about how learning cultures are influencing the use of technologies and vice versa how the introduction of these technologies shapes innovative learning cultures in VET.

## Introduction

*Digital change will arrive, also at workplaces, how we cope with change will be important in the future*. (Workplace trainer, login)

Workplace learning in the context of vocational education and training (VET) in Switzerland takes place in companies, VET schools and training centres. The major part of the apprenticeship is practical learning facilitated by companies and they are the most important pillar within the VET system (Gonon 2007). Often innovations in learning are transferred from workplaces into schools (Pfeiffer 2015). For enterprises, the ways in which they develop young adults for the world of work are crucial, not least to maintain firm competitiveness, because vocationally educated employees are the backbone of the economy (Häfeli et al. 2015; Finegold and Wagner 1997).

Switzerland’s large majority of young adults between 15 and 17 years old (about 70% of each cohort) enrol in VET (SBFI 2019). They earn a salary that increases over the 3–4 years of apprenticeship training until the Federal VET Diploma is acquired. The dual structure of training, which can be found in Germany and Austria as well, provides early labour market experience and employment opportunities after graduation (Wettstein and Gonon 2009). Usually, hiring apprentices pays off for enterprises based on the productive work that apprentices are able to provide (Wettstein and Gonon 2009; Schweri 2019). Companies and labour market organizations profit from providing career prospects for young people, because this secures the supply of skilled workers needed in a branch (Rupietta and Backes-Gellner 2019; SBFI 2017).

While domain-specific knowledge and skills are highly relevant and need to be acquired during apprenticeships, the relevance of personal competences, such as creativity, taking initiative and working autonomously, is increasing (Barabasch and Keller 2019a). Self-organization of work is more and more requested of workers and needs to be trained early. Regulation and control activities that originally were in the responsibility of management are handed over to workers (Heinz 2009). According to Filliettaz (2010), the organization of workplaces is a decisive factor for enabling workplace learning. Usually, learning is strong when the content is challenging, if the employees can take over responsibility and increasingly self-organize their work, and if they are hereby adequately supported (Nyhan 2009). Innovation-oriented enterprises do adapt their VET training according to these requests, for example providing flexibility and individuality in workplace training, project-based learning or new forms of learning accompaniment.

The use of digital technologies in vocational education and training (VET) in Switzerland has been reinforced by the 2020 Corona crisis. Although, education at all levels and research had strongly focused on a wide variety of issues related to digitalization (SBFI 2020), the requirement to competently work with a number of digital tools became even stronger now. Digital technology integration or adoption has become crucial not only for communication, administration and management, but it is also a meaningful asset to support learning and teaching in VET. Modern learning cultures take approaches to successfully integrate technologies in their internal processes, and in this way support autonomy and flexibility in work and learning, lifelong learning as much as intergenerational learning. They further contribute to connecting different learning sites, such as school, workplace and intercompany-training course (branch-course). While digital technology is promising to facilitate such connections, today, the facilitation of the connection of the learning sites by digital technologies is not a general standard (Cattaneo and Aprea 2018; Schwendimann et al. 2018).

The enterprises Swisscom, Login and Post in Switzerland have integrated various digital technologies in their apprenticeship training. These technologies ease administration of work hours, work tasks, evaluations or log book remarks; they support communication with peers, co-workers and trainers; and they come in handy for idea development and creative work. Overarching trends in terms of changing learning cultures in apprenticeship training, such as individualization, flexibilization, self-organized learning, project work or coaching, support the introduction of these technologies and also benefit from them (Barabasch and Keller 2019a, b; Barabasch et al. 2019). Based on three in-depth case studies in these three Swiss enterprises (Yin 2014), results on the usage and impact of digital technologies will be presented. Data were collected by means of semi-structured interviews with apprentices, workplace trainers, coaches and VET management.

This chapter addresses the following research questions: Which digital tools are used in workplace training? What are potential benefits and extended justifications for the use of digital tools? How are modern learning cultures impacting the use of digital tools? We will outline how and where digital technologies are used at the workplace in apprenticeship training, why management has introduced them and how apprentices and their trainers benefit from it. Based on our findings, we will draw conclusions about how learning cultures are influencing the use of technologies and vice versa how the introduction of these technologies shapes innovative learning cultures in VET.

## Theoretical Foundations

The use of digital technologies shapes individual as well as work-related learning and competence development and plays an important role in VET. Next to the introduction of digital technologies in formal learning, increasingly, informal learning becomes an incremental feature of digitalized work (Dehnbostel 2020). Especially, the current reinforced policy to work at one’s home office may support this fusion of different life spheres. Not least, the increasing amount of mobile technologies being available enables the more flexible use of time and space (Tubin 2006). However, the flexibilization of work itself as much as the extensive work with technologies may cause various work-related troubles, such as various health and psychological problems.

 Digital technologies usually rise up in different contexts and for different aims that are not necessarily educational ones. Most of the times, they are then adapted for educational purposes (Januszewski and Molenda 2008). At this time, digital technologies have entered workplace learning in many different forms, especially as production or design tools (e.g. numerical control machines, electronic measurement devices and computer-aided design software), but their use as a training tool within VET remains under-exploited. The usage of media in vocational education and training can take various forms, for example, the usage of presentation media, exchanges among students in group learning or self-study in digital learning environments (Euler and Wilbers 2020). A quite common use of technologies is to develop learning platforms or collaborative online learning spaces (Sonntag et al. 2004; Willey and Gardner 2012). Within these environments, apprentices can experience, practice, reflect and improve their ability to work with various forms of learning.

They further open up opportunities for new ways of designing and enacting learning. Research focused on location-based (or place-based) learning (Jones et al. 2013), “all the time, everywhere” learning (Norris and Soloway 2013), learning ‘on the move’ (Sharples 2013) and in “multiple contexts” (Mifsud 2014) (see Schuck et al. 2017). Especially using handheld devices to explore “seamless learning” tasks (Hedberg and Stevenson 2014; Rushby 2012; Toh et al. 2013) has been intensely explored, because of its possibility to support a transition of learning across contexts, often between formal and informal learning spaces. Of interest has been the “breaking away from text, time and place” (Hedberg and Stevenson 2014, p.17), connecting learning in and out of class, in and out of school, connecting learning across curricular and extra-curricular activities, learning that is social or personal, academic or recreational, that exists in physical or virtual contexts and across times and locations (Wong and Looi 2011). “Seamless learning” helps to connect learning between school and excursion sites and provides a bridge between classroom-based tasks and more realistic fieldwork settings, or providing a transition from a personal, informal learning episode at home to learning at a later time at school.

Using digital technologies may support more independent or autonomous learning as much as cooperative learning. It is important that a general learning ability is established within apprentices so that lifelong learning is easier and individuals are inclined to keep up with new innovations (Schüller-Zwierlein and Stang 2011). Teachers are working more in the background, are less concerned with teaching domain-specific knowledge, but rather advise and coach individually (Kozma and McGhee 2003; Mandl et al. 2003; Schulz-Zander and Riegas-Staackmann 2004; Schulz-Zander 2005).

A change in learning-related values and norms can already be observed (Sonntag et al. 2004). Digital natives are quite familiar with a variety of digital tools (Prensky 2005) and develop different habits to work with them. In VET, this changing behaviour of learning on- and offline makes it easier but also advisable to accommodate teaching, workplace coaching and learning with digital technologies. This paradigm shift is an important characteristic of innovative learning cultures.

The term learning culture can be used to characterize the embeddedness of learning in the interaction among context, concept and reality (Brown et al. 1989), which refers to learning as truly being embedded within cultural settings and the utilization of cultural resources (Bruner 1996, as cited in Hodkinson and James 2003). Components of a learning culture include the learning environments as well as the practices and procedures of working and learning. In addition, it includes the study of attitudes, values and beliefs among the practitioners involved in training and of the apprentices themselves. Both are constantly influencing each other.

Culture further consists of variables, such as values, beliefs and attitudes that are common within a community and tend to perpetuate themselves, sometimes over long periods of time. It includes collective memories, long-held assumptions, common expectations and definitions (Ai-Tzu 2015). In an enterprise context, according to Sonntag et al. (2004), the learning culture is an expression of the importance of learning within the enterprise, which targets the development of competences and innovation. The learning culture is shaping individual, group- and organizational learning processes in connection with relevant framework conditions.

To summarize findings: At a normative level, learning culture is expressed through values, norms and attitudes related to learning. At the strategic level, learning culture is related to framework conditions and support for lifelong learning. At the operational level, learning cultures find their expression within the manifold forms of individual, group-based learning and organizational learning. Learning cultures are an orientation for the members of the organization in providing expectations towards the results of learning and they can be actively shaped (Barabasch et al. 2020a). Considering the usage of technologies at the normative level, questions about the ways in which technologies are used may be raised. At the strategic level, the concern lays on work and learning conditions in respect to the usage of technologies, and at the operational level, one can analyse how technologies are used or adapted in various learning settings. All of it is not necessarily prescribed by an organization, but within innovative learning cultures would rather be shaped by its members, including the apprentices.

## Method

To research the usage of digital tools in VET, three case studies (Yin 2014; Yin and Davis 2007) in Swiss enterprises that train apprentices in an innovative manner have been conducted. The three enterprises represent different sectors of the economy. The first one operates in the telecommunication industry. In Switzerland, it is the largest provider for traditional telecommunication services as well as in the provision of software solutions. The second enterprise provides VET training for apprentices that work in the public transportation sector. It cooperates with 50 partner-enterprises of the sector, for which they organize their VET training. The third enterprise is the national postal service, which entails also the two departments finance and transportation (bus).

Participants in the case studies represent the main stakeholders in workplace training at the three enterprises: Apprentices, workplace trainers, personnel that directly works with apprentices such as coaches, as well as persons representing different levels of VET management. The main data source were semi-structured interviews with persons representing all groups of people involved in workplace training (case one 25, case two 60, case three 60). Furthermore, site visits at different working (and learning) venues were conducted (case one 7, case two 18, case three 18). Data collection was completed by document analysis of VET-related documents of the enterprises. Participants for the interviews and locations for site visits were selected by the team of researchers together with a VET manager at each enterprise. The cooperation in the selection of interview partners led to a flexible continuing enlargement of the sampling in a function of theoretical sampling, leading to data saturation, respectively, to a profound understanding of the cases. The interviews followed a general interview guideline aimed at finding out about daily work, regular tasks, successes and difficulties, the organization of VET programmes, support by workplace trainers, as well as attitudes, values and beliefs regarding the workplace training. Data were analyzed by a content analysis (Kuckartz 2016). Two coders coded the entire material, supported by the software MAXQDA. The material was structured according to individual cases and categories representing different research topics (Kuckartz 2016). In an iterative process, the narratives were coded according to emerging themes and regularly discussed by the research team to ensure the reliability and validity of the data. In this way, a comprehensive and detailed system of categories was derived. The analysis of the coded segments led to a display of how digital tools are used in the workplace training at the three workplaces.

## Findings

The enterprises Swisscom, Login and Post in Switzerland have integrated different digital technologies in their apprenticeship training in order to facilitate processes of workplace training. Some tools used in the specific learning and working environment of the different enterprises are developed for the training of apprentices, others are adapted to meet the specific needs in this respect. Due to large numbers of apprentices, developing digital tools for training is an economic and valuable option for these enterprises. They became particularly useful throughout the Corona crisis. All three enterprises have managed to change their working modus to digital work and home office within a few days. Login was able to move from classroom instruction to digital lessons within 2 days. Students responded very positively to it, so that the enterprise is planning to have more digital instruction in the future. The following section will first summarize the tools used in the three enterprises, then report on the experiences using communication tools, and in the third section, we explore three particular benefits of using digital tools that emerged from our data.

### Digital Tools Embedded in the Specific Work and Training Structures

Within the Swisscom learning environment, the tool “market place” has been specifically developed for training and is a vital element of the workplaces learning culture in VET (also see Barabasch and Caldart 2019; Barabasch and Keller 2019a, b; Barabasch et al. 2019, 2020b; Keller and Barabasch 2019). Apprentices at Swisscom do not work together solely with one workplace trainer or only one internal department over the duration of their apprenticeship, as this can be the structure in other Swiss apprenticeships, but instead, they work and learn in different projects. All departments, where workforce is needed, can advertise projects for apprentices on the market place. In this way, the workforce of apprentices can flexibly be integrated where there is a need for them, an organization of workplace training that fits well with todays’ fast-changing workplaces (e.g. frequent organizational and personal changes) in dynamic industries, such as the telecommunication sector (Barabasch and Caldart 2019). Today, according to VET managers of the enterprise, the market place is not only used in the VET training but serves as inspiration and is used for work organization (distribution of tasks) also for regular workers on other levels. Next to this organizational tool, the tool “eNEX” serves as a platform for the documentation of the competence development of apprentices and provides an overview about development progress, which is the base for the interaction with coaches. It further is a navigation tool for projects, that accommodate the acquisition of competences as outlined in the requirements of the training ordinance.

Login is a training provider for apprentices of the transportation sector . Apprentices work and learn at different partner firms to develop skills in various contexts. The training enterprise hires apprentices and manages all main organizational tasks, such as communication with vocational schools or monitoring the overall development of apprentices. They also provide additional courses for developing specific competences needed in the transportation sector. At the partner firms, workplace trainers supervise and accompany apprentices by working with them on a daily basis. Partner firms profit from this organization, they have VET training outsourced to some extent, but still give the apprentices and possible future workers in their firm the possibility to gather work experience in a real workplace environment. Of course, they also profit from apprentices’ productive work-outcome (also see Barabasch and Keller 2020). The organization of courses for the different occupations, the changes of workplaces between different partner firms and the accompaniment by different workplace trainers are quite complex to oversee. Login uses the tool “time2learn,” which helps to cope with this complexity. It is used to document the learning progress of the apprentices and their school grades and manage their course planning, which they discuss and oversee together with their trainers. The tool is also used for communication with trainers and access to course content.

Apprenticeships at Post do not follow a common structure, but instead there are different designs and logics in the various programs. For example, in some occupations, apprentices internally change workplaces during their apprenticeship and get to know different departments of the firm; in others, the apprentices remain in one department. For some apprenticeships, it is possible to work at another enterprise for some months, to acquire certain competences, that cannot be developed internally; in other apprenticeships, it is foreseen that apprentices travel abroad for some weeks, to enhance their language skills (see Barabasch and Keller 2020).

The structural organization of training at Post calls for a specific selection and development of tools. A didactic model has been developed internally and is continuously adjusted. It builds the foundation for training courses within the enterprise aiming at a digitalization of learning. Some of the courses are already entirely digitalized; other courses remain analogue, as requested by the ordinances and curricula (the legal basis of the structure of apprenticeship training in Switzerland). The main learning platform is “Moodle.” It provides access to online courses and needed digital materials and tools, but also lists presence courses, such as compliance courses. If an apprentice starts his/her apprenticeship, he/she is assigned to a class and receives an overview over the 3–4 years of training, views dates, rooms, as well as “nuggets,” modules of self-study and presence-modules. There are also modules for the acquisition of additional competences, for example, in the field of leadership or project management.

The central documentation of information is one of the main reasons that digital tools are used in VET in the researched enterprises. Usually, a member of human resources (e.g. coach or trainer) advises a group of apprentices, while for the technical training, apprentices work with (different) specialists in the field. Digital tools, such as the mentioned platform “time2learn” or “eNEX,” provide the possibility to oversee learning progress among the apprentices. With the overview provided online, supervisors can react quickly, if unforeseen developments occur or if a project manager or a specialist working with apprentices reports a problem.

Next to these major platforms, each enterprise operates with a number of applications throughout their apprenticeships. Apprentices use “Real Time Management RTM, SAP” to report working hours and absences or survey tools, such as “Forms” or “360 Feedback.” “Office 365” is frequently used, with programs, such as “Word,” “Excel,” “OneNote,” “SharePoint,” “Planner” and “PowerPoint,” for data storage, exchange of information and planning purposes. The following table provides an overview about the most common digital tools used in apprenticeships at the three enterprises (Table 7.1).

**Table 7.1:** Digital tools used at Swisscom, Login and Post in Switzerland

Swisscom	Login	Post
Marketplace eNEX Word Excel Power point Outlook Mail & Calendar Teams (chat function in slack today has replaced slack, which was earlier in use) Planer OneNote OneDrive SharePoint Skype for business Telepresence-rooms https://ch.linkedin.com/learning MyImpact MyContribution Microsoft forms	time2learn (sometimes also Konvink) Real Time Management, RTM Word Excel PowerPoint Outlook mail & calendar Teams OneNote Yammer Planner SharePoint	Moodle SAP solutions Word Excel PowerPoint Outlook mail & calendar SharePoint Confluence Starmind Skype (for business) Telepresence-rooms 360 feedback Azure Defops Jira Status meeting tool Wiki

Internal IT departments are keeping up these tools, take care of upgrades and of data security. The latter can be a constraint for the usage of certain tools. For example, in the enterprise Post, the VET department is part of the human resource department and due to the sensitivity of the information processed and issues around data protection related to “Teams,” this program cannot be used there. A member of the organization of the training for ICT apprentices stated:

*We would really like to include them (the apprentices) in using Teams, but until know, this is not possible, because Teams is out in the cloud… That’s difficult in our department regarding collaboration* (VET manager ICT, Post).

The example shows how internal organizational processes are not digitalized due to the lack of data safeguarding. The challenge may prevent the theoretical possible ease of communication and collaboration expected by the usage of these tools.

Above and beyond these internal complications, when it comes to the collaboration between vocational schools and enterprises via digital tools, developments are slow. Too often, information on absences of students or behavioural issues are reported in paper booklets, which apprentices, trainers and teachers have to sign. The organization and usage of digital tools is either a question of individual schools or the organization of the canton in Switzerland. While it can be expected that the current Covid-19 crisis may speed up developments, the likelihood of enterprises reaching out to schools in their interest to ease processes, is just as high.

### Tools Easing Communication in a Modern Learning Culture

 Digital technologies facilitate and structure forms of communication in the enterprises. Chat functions are used for rapid informal exchanges (“WhatsApp”) among apprentices and between apprentices and their coaches or trainers, or for official communication (“Skype for Business,” “Teams”). Emails (“Outlook”) are still used, although participants at Swisscom state that mail is continuously being replaced internally by “Teams.” Call and video tools (Skype or “telepresence” rooms) enable conferences and help to safe on travelling. In “telepresence rooms,” the communication resembles face-to-face interaction due to the use of large displays, differentiated cameras and high-end microphones. It also became obvious that there are no enforced restrictions as to which tools need to be used for communication. Apprentices can flexibly contact their trainers and coaches via phone, email or just placing an appointment for a coffee break in their calendar. Due to these spontaneous interactions, trainers and coaches can react timely and provide the support needed. However, for the trainers, the communication with different tools can be challenging, since one needs to keep track of the communications and requests on the different channels*.*

*I use the phone much more than two, three years before… and there are different channels. There is SMS, WhatsApp, than we have Slack- that’s another channel through which we communicate, where I have a group chat with the apprentices, about different themes. And, that is a new challenge. In the sense, that I am “bombarded” on different channels and have to handle that. When questions arrive… I sometimes don’t remember, on which channel was that again? Where did I read that?* (Coach, Swisscom)

If apprentices have difficulties and need support, they can easily get in touch with individuals of their choice, such as other apprentices, experts in certain fields, external business contacts or coaches. In the enterprise Post, apprentices (as well as regular workers) use “Starmind,” where open questions can be placed “in the cloud.” This makes it visible for the entire network and is assigned to the department, which thematically best fits the question. The members of the corresponding department may further assign the question to a specialist who can answer it. In the enterprise Swisscom, employees have internal profiles, on which their competences are displayed and on which it is also visible, with which technologies he/she works. Apprentices, as well as regular workers can contact them, if needed.

In todays’ workplaces, networking is a central element for success. Tools such as Yammer or Teams allow to establish one’s own network in the firm. Building groups for exchanges regarding technologies and for the organization of project work is, for example, also possible on the portals “Azure Defops” or “Jira,” which are used for software development at the enterprise Post.

*WhatsApp and WhatsApp class- chats are popular. Everything else often takes too much time: opening the laptop, going to a certain website, and then again opening the chat-tool on this website… everyone has a smart phone on hand, everyone responds immediately, that’s easier!* (Apprentice, Post)

Some communication tools are also questioned in terms of data protection, especially “WhatsApp.” Whether a tool is useful or not lies in its practicality; tools have no value in themselves. At different workplaces, it was reported that communication platforms have been established and then not used as much as expected, while other informal tools are commonly used by apprentices. Also, having a tool readily available on the smartphone increases the chances of being used by apprentices.

Apprentices, during their apprenticeship, are prepared to communicate, work and learn with digital technologies, for example using E-Learning and Web-Based Training in internal courses. Enterprises are also rolling out administration and organization of workplace training digitally as far as possible. Learning to work with digital technologies may take place “spontaneously,” since apprentices learn to work with new technologies while working in an increasingly digitalized world of work. Effects of digitalization can be seen in almost every occupation, for which an apprenticeship is available in the three enterprises.

Apprentices in ICT occupations are especially confronted with high innovation dynamics and often in their work use (the always changing) newest technologies. They need to be highly adaptable and open. Also working with customers in various fields requires the development of digital skills. At train stations, for example, customers are accompanied in buying tickets on their mobile phones. In branches of the Post- Bank, customers are introduced to how to use online banking tools.

The internet as well as online research and learning is frequently used at Swisscom. In some cases, workplace trainers advise apprentices on the following issues: which online tutorials to learn from, how to access relevant sites and courses and which sites to use for further information. In the department of ICT training at Swisscom, there are, for example, several lists of useful links available through which further information can be easily accessed. Besides that, autonomous research to find solutions and missing information to work with new technologies is a popular way of learning and also expected from apprentices.

*So, independent does not actually mean that one does everything on ones’ own, it just means that one can acquire knowledge on ones’ own. In the sense, that I can acquire more knowledge through internet resources, or that I independently approach people. And also, in some cases talk with people that I don’t know, and look at the topic with them or just ask them.* (Apprentice, Swisscom)

Many interviewees reported that there is a difference in how easily change is coped with: It differs from generation to generation. Apprentices are considered to be open to use new tools and also bring in their creative ideas on how work processes can be facilitated or how tools could be optimized. Digitalization does not only bring about new tools but also calls for new processes, new ways of thinking and a different organization of work. As to some extent older employees struggle with changes, at Swisscom, apprentices may function as “ambassadors.” They provide tutorials to older employees.

What also became evident in the interviews is that the impulse to consider “digital natives” as competent users or even developers of software is often not matching reality. They are indeed at ease using their mobile phones and navigating through social networks, but this does not mean that they do know, for example, how to use Office components. There are a large variety of competences that someone needs to have to work with computers and while some apprentices start with little prior knowledge and need to learn a lot, others have had experience, but need to build up extended knowledge and expertise.

### How Learning and Competence Development Change

Working with digital tools throughout their apprenticeships offers a number of advantages. Among them is a higher flexibility in terms of time and space. Many apprentices, especially in the fields of informatics and mediamatics, in the three firms have flexible working hours; some also have the opportunity to work at different company locations, in co-working spaces, hubs or even from home. This supports their autonomy in the way they organize themselves, requests from them to work independently, structured and self-organized and manage their flexibility wisely to be productive.

*My boss tells me what he expects from me, what my main tasks are. Then, I have to look for what I need by myself and have to search for more information. We are completely free and can think for ourselves, ‘how do we get as fast as possible to the solution that we finally need?’ And then, we have the different aids that we can use. Of course, there are the internal tools that help us. One is for example Skype for Business, with which working together and being mobile is made easy. Also, with the laptop that we receive, this enhances our mobility and I have the opportunity to work from home, for example. And, there my boss really says: ‘Look, you have this time-span, and you have to work on this project during this time-span. How you do it, I leave it up to you. The result just has to be right.’* (Apprentice, Swisscom)

Flexibility also accounts for individuals’ adjustment to constantly changing tools. Apprentices realize throughout their training that their attitude of openness is vital to successfully work in this work environment, because it requires a constant updating of one’s skills and competences. Lifelong learning has become a panacea for successfully working with digital tools, which in itself is the precondition for apprentices’ adjustment to the new organization of work. Constant software and hardware changes, but also changes in work processes, make apprentices realize how important constant learning and new skills acquisition is.

*Of course, to be successful, one has to believe in it. One should not go to work and say: ‘Yes, another workday, like the others, like the one before’. One has to be always curious. Every day one has to be attentive to news… because Swisscom, for example the sector of mobile telecommunication is a huge sector, every day there are new developments… One has to be curious, one has to inform oneself.* (Apprentice Swisscom)

*The apprentices are aware, that this targeted acquisition of knowledge does not end with graduating from the apprenticeship, but that they have to continue to learn. This attitude is specifically important under todays’ premise of lifelong learning. The apprentices develop the perspective, already throughout their apprenticeship, that the acquisition of domain specific knowledge is a continuous process, because the working world constantly changes.* (Apprentice Swisscom)

Although, technologies are changing fast and updating oneself is a given precondition for working successfully, the apprentices realize some intergenerational differences. Openness, attitudes towards change, approaches to work and work organization and the usage of new technology often differ between older and younger employees in the company. Some older employees struggle to keep up with technology developments and are supported by apprentices. There are apprentices who prepare tutorials, provide instruction or individually accompany older employees to help them understand new technological tools. Intergenerational learning and teaching can be an aspect of a project at Swisscom.

*Older employees, or people that work since a longer time, they look at it from a very different perspective than the young. For the young, technology is extremely important, and how one can do things quickly. The older try to do it so it’s really nicely and completely done… they look at things from one step to another. Young people always try to find short ways, so that it works more quickly, so that one can do it in a way that saves time. Older people prefer to make a step more, in the way, they are used to do it from the beginning.* (Apprentice login)

*I think the older generation profits from the fact, that the young can explain them how today’s technologies function. I think they do not have a big problem with that. Maybe they rather have troubles with the fact that what they have known earlier increasingly is pushed in the background. But besides that, I think they are really happy if the young can help them with these things.* (Apprentice login)

Digital technologies not only provide the opportunity to work or communicate more efficiently, they also change the ways in which apprentices learn and work. While they are supportive of autonomous work and enable a flexible use of time and space, they also require constant learning and updating of one’s skills. Since the young generation tends to be faster in learning in this respect, intergenerational learning lets older employees benefit from it. This trend also contributes to a change in how apprentices are integrated at the workplace and questions traditional ways of viewing the development of expertise.

## Conclusion

Modern learning cultures in apprenticeship training are characterized by an individualization of learning pathways, more autonomous work often supported by digital tools, new team work organizations, new approaches to teaching and learning and trustful relationships between apprentices and their supervisors, coaches or trainers. Enterprises in Switzerland have made it a commodity to work with a large variety of technological tools to ease learning and work, an asset that helped them to cope successfully with the move to home office during the Corona crisis. Many of the tools are particularly helpful in easing communication within teams, across locations and in different work settings. Apprentices are often quick in learning how to use these tools and do help older employees in understanding and mastering them as well.

When it comes to technology learning, the novice-to-expert paradigm (Dreyfus and Dreyfus 1987) seems to be reversed. Fast technological developments enforce the need for lifelong learning, which apprentices are aware of. For many of them, undertaking an apprenticeship is the first step into working life, but the willingness to engage in further vocational education and training as much as in a variety of options for adult education is high among young people. They know that innovation requires a confident handling of technologies and a disciplined self-organization. The Corona crisis certainly has put a test to that. It strongly indicated that the much conjured digital transformation has taken place for many in VET suddenly and rapidly. Within VET from now on, it will be about manifesting the chosen pathways, further qualifying teachers and trainers and to establish a learning culture that accommodates new approaches to teaching and learning at VET schools and at enterprises. We are at the rise of a major change process that will involve all actors and be an intergenerational learning process (also see Heinen and Kerres 2017).

The new learning culture requires a new understanding of roles in terms of teachers and apprentices, where both can learn from each other and interact in many new forms. Coaching and advising students in their learning process will become more relevant in order to help apprentices to individually navigate their learning process and grow from making mistakes when curiously trying out new things. Digital technologies may support this pathway as much as they support new forms of collaborative team work. While they make travelling time less necessary, they may also support a new balance of life and work.

Based on the findings from the three case studies, it becomes evident that enterprises are encouraging the use of technologies at all levels. VET schools are also responsive to digital trends and need to quickly learn how to work with different tools due to the Corona crisis. Further research is required to investigate the state of teacher preparation for working with new tools, new approaches to teaching and learning as well as to connect the learning between different learning sites. For enterprises, this chapter may provide an up-to-date overview about the tools currently used and how they can be applied within workplace training. Considering that intergenerational learning becomes especially relevant in working with technology, a learning organization should facilitate this in formal ways. More research is needed to fully understand how the majority of enterprise-based learning places respond to digital change and which lessons can be learned from that for VET schools.
